# Simpler grammar, larger vocabulary: How population size affects language

**DOI:** 10.1098/rspb.2017.2586

**Published:** 2018-01-24

**Authors:** Florencia Reali, Nick Chater, Morten H. Christiansen

**Affiliations:** 1 Department of Psychology, Universidad de los Andes, G230, Cra. 1 Nro. 18A-12, Bogotá 11001000, Colombia; 2 Behavioural Science Group, Warwick Business School, University of Warwick, Coventry CV4 7AL, UK; 3 Department of Psychology, Cornell University, Uris Hall, Ithaca, NY 14853, USA; 4 The Interacting Minds Centre and School for Culture and Communication, Aarhus University, 8000 Aarhus, Denmark

**Keywords:** cultural evolution, language change, social structure, population size, language complexity

## Abstract

Languages with many speakers tend to be structurally simple while small communities sometimes develop languages with great structural complexity. Paradoxically, the opposite pattern appears to be observed for non-structural properties of language such as vocabulary size. These apparently opposite patterns pose a challenge for theories of language change and evolution. We use computational simulations to show that this inverse pattern can depend on a single factor: ease of diffusion through the population. A population of interacting agents was arranged on a network, passing linguistic conventions to one another along network links. Agents can invent new conventions, or replicate conventions that they have previously generated themselves or learned from other agents. Linguistic conventions are either *Easy* or *Hard* to diffuse, depending on how many times an agent needs to encounter a convention to learn it. In large groups, only linguistic conventions that are easy to learn, such as words, tend to proliferate, whereas small groups where everyone talks to everyone else allow for more complex conventions, like grammatical regularities, to be maintained. Our simulations thus suggest that language, and possibly other aspects of culture, may become simpler at the structural level as our world becomes increasingly interconnected.

## 1. Introduction

It has often been observed [1–4] that the properties of human languages appear to be influenced by the size and degree of isolation of the linguistic community. Small, isolated linguistic communities often develop languages with great structural complexity, elaborate and opaque morphology, rich patterns of agreement and many irregularities [1–5], and it has been argued that such ‘mature’ features of languages require long interactions in small, close-knit societies [6–8]. By contrast, languages with large communities of speakers, such as Mandarin or English, appear to be structurally simpler. Language compositionality has been shown to be inversely correlated to irregularities and nonlinear morphology [3]: regular languages are more frequent in large-sized communities, while irregular, morphologically complex languages tend to arise in small-sized ones. Computer simulations have shown that linguistically ‘marked’, and hence complex, patterns arise more easily in small populations [9,10] and that compositional structures tend to emerge more extensively for larger groups [11]. The causal role of the size of the linguistic community is, moreover, further indicated by the historical tendency towards structural simplification as a language gains an ever-larger community of speakers [12].

But an apparently opposite pattern appears to be observed in relation to non-structural properties language: languages with large linguistic communities tend to have larger vocabularies of content words. For example, the vocabulary of wide-spread languages, such as English, appears to have grown rapidly in historical times, and is typically estimated to have many hundreds of thousands of words, including those with highly specialized and technical meanings [13]. Despite their frequent spectacular structural complexity, languages spoken by small bands of hunter–gatherers are typically assumed to have smaller vocabularies, although reliable data for such languages are difficult to gather [14]. An analysis of Polynesian languages indicates, moreover, that larger linguistic communities both create more new words and lose fewer existing words over time [15]. These contrasting patterns pose a challenge for theories based on the cultural evolution of language. Recently, theorists have suggested the erosion of complexity in larger language communities arises from the greater proportion of second language learners [1,16]. But why do such arguments for simplification not also apply to the lexicon?

One possibility is that structural and lexical aspects of language might diffuse through different mechanisms. For example, adult–child interactions might be the primary vehicle for regularizing morphology or syntax (see [17,18] for contrasting perspectives) and adult–adult interactions might be the primary vehicle for lexical innovations. Moreover, there may be differential impacts of language contact on structural and lexical aspects of language: lexical items diffuse across languages more readily [19]. Such effects might be amplified to the extent that structural and lexical aspects of language share a fixed communicative burden, so that, for example, simple morphology must be compensated by a larger vocabulary.

While such factors may play a role, we focus on a more parsimonious alternative: that the opposite relationships between population size and lexical versus structural complexity depend on a single parameter: ease of diffusion. Structural aspects of language diffuse slowly because they are difficult to learn, are absorbed slowly and piecemeal by first language learners, and often present persistent challenges for second language learners [20]. Words, by contrast, can often be acquired from just a few exposures [21]. An account based on ease-of-learning suggests that an increasing population of speakers should, even within the category of words, lead to an increasing prevalence of easy-to-learn words (e.g. concrete words) over hard-to-learn words (e.g. abstract words). A recent corpus analysis of two centuries of American English does, indeed, show an increasing proportion of concrete words [22].

To illustrate this scenario, we divide properties of language, as a first approximation, into two basic categories—*Easy* and *Hard*—requiring, respectively, few or many exposures to be acquired by a new speaker. Easy properties of the language can rapidly be transmitted across the linguistic community. As the community grows in size, so does the number of members who can spontaneously modify or invent new Easy properties (such as lexical items) that can diffuse across the community. Hence, large communities will end up with large inventories of Easy features. Conversely, in large linguistic communities, speakers will have minimal interactions with many other speakers, so that typical interactions between individuals will be too limited to transmit the Hard linguistic property successfully.

If correct, this simple mechanism should apply to cultural evolution more broadly. Indeed, new and structurally complex, and difficult to acquire, cultural forms develop in small, tight-knit communities who interact intensely, as in the birth of bebop in 1940s New York [23], or the lindy hop at the Savoy Ballroom in 1930s Harlem [24]. By contrast, mass cultural forms tend to be structurally simple and easily learned. For example, we are now exposed to, and recall, a huge number of popular tunes; but most are harmonically and melodically simple; and statistical analysis suggests that modern popular music appears to be gradually getting simpler over the decades [25].

Can these intuitions be made precise by computer simulation? Building on prior preliminary work [26], we created a novel innovate-and-propagate (IAP) process, operating over populations of simulated agents. Agents are arranged on a network, so that agents connected by a link on the network can ‘converse’ and hence, potentially pass linguistic ‘conventions’ to one other. Each agent is not only able to ‘invent’ entirely new conventions but can also replicate conventions that they have previously generated themselves or learned from other agents (i.e. agents to which they are connected by links in the network). When an agent produces a convention (whether novel or a replication), it propagates that convention to one of its neighbours.

Our simulations show that the size of the network can potentially have opposite effects on the richness of different aspects of the language. A simple quantitative change—the ease of learning of an item—responds qualitatively in entirely different ways to population size. Linguistic innovations that are relatively easy to learn (such as new lexical items or modifications to existing ones) increase in number as a linguistic community grows, because the number of potential innovators increases and innovations can spread more rapidly. By contrast, small linguistic communities favour linguistic innovations that are hard to learn (such as, we suggest, structural changes in the language), because they require multiple interactions between individual speakers for their continued existence.

## 2. Simulations

### (a) The model

To capture the dynamics of individuals interacting with one another, either conversing by way of old conventions or inventing new ones, we use a modified version of the Chinese restaurant process [27], which we call the IAP process. The Chinese restaurant process is a widely used probabilistic model defining the frequency distribution over a potentially limitless number of types (e.g. linguistic conventions, words, categories). It embodies the assumption that the ‘rich-get-richer’—the probability of a token of an existing type is proportional to its current frequency (i.e. the chance of the new diner sitting down at a given table is proportional to the number of diners already at that table), while also allowing the creation of new types (i.e. a diner being seated at a previously unoccupied table).

In our extension to the IAP, we view each agent as corresponding to a ‘restaurant’ with a finite, but infinitely extendable, number of ‘tables’, i.e. conventions. Each time the agent generates a convention, it chooses an existing convention with a probability proportional to the number of previous tokens of that convention; this is equivalent to seating each new customer in the restaurant at a table in proportion to the number of customers already seated at that table. But it is also possible that an entirely novel convention will be generated (a new table in the restaurant is created, and the new customer becomes the first person sitting at that table). This occurs with probability 1/(*M +* 1) (where *M* is the number of current restaurant customers – stored tokens).

As described thus far, each agent generates conventions entirely independently, not sharing those conventions with the rest of the linguistic community. IAP introduces a simple extension of the Chinese restaurant process to deal with this. At each iteration, every agent ‘utters’ a convention and passes it to a randomly chosen immediate neighbour. For each agent, the probability of generating an existing convention is determined by the sum of the number of times that it has, itself, previously generated that convention added to the sum of the number of times it has received that convention from an immediately neighbouring agent (provided the agent has already learnt that convention). Thus, in this model, agents tend not merely to generate what they have generated before; but also to generate what they have ‘heard’ (and learned) from neighbouring agents.

As the simulation progresses, agents will invent conventions and pass them on to each other. Thus, initially the number of conventions used by the agents (i.e. the complexity of the language) will gradually increase. However, the number of conventions is limited by restrictions in cultural transmission. Two versions of information transmissions are implemented. In the horizontal transmission version, conventions are passed among immortal but forgetful peers. Each time an agent picks a convention (new or old), then, for each of the M convention tokens that it currently stores, there is a probability that this token is forgotten. In the vertical transmission version, peers eventually ‘die-off’ (their convention repertoire disappearing with them) and are replaced by new peers who are initially ‘blank slates’.

So far, we have not distinguished between Easy conventions (which can be learned from another agent by minimal exposure—these correspond to lexical items) and Hard conventions (which require multiple exposures—these correspond to structural properties of the language). To get started, we make the simplest of distinctions between them: Easy conventions can be learned by an agent from a *single* exposure. Once a convention has been generated by a neighbour, an agent can immediately generate that convention. Hard conventions can only be learned from *two* exposures: only when an agent has encountered two examples of the exact same convention from its neighbours (whether from the same or different neighbour), will this convention be seated at a new table (representing that convention in the agent).

### (b) Networks

Agents are represented as nodes in a non-directed graph (one in which edges have no orientation) and links between neighbouring agents are represented by edges between nodes. Networks are characterized by three parameters: *n* is the number of nodes (i.e. the population size), *k* is the mean nodal degree (i.e. the number of links (neighbours) that an agent can communicate with, averaged across agents) and *C* is the clustering coefficient (i.e. a measure of the degree to which nodes (agents) in a graph tend to cluster together).

The structure of our networks is inspired by real social networks, based on recent work finding quantitative relations between city size and the structure of human interaction networks from mobile communication records in Portugal and the UK [28]. Mobile phone communication has been argued to be a reliable proxy for the strength of individual-based social interactions [29]. The results in [28] revealed that the number of average contacts per mobile phone (nodal degree, *k*) grows superlinearly with city population size, according to the well-defined scaling relation: *k* ∼ *n^β^*^−1^, where *n* is population size. These results fit prior theoretical work suggesting that superlinear scaling stems from the nature of human interactions [30]. Interestingly, the probability that an individual's contacts are also connected with each other—i.e. the clustering coefficient *C*—remained constant (*C* ∼ 0.25) across city sizes [28].

### (c) Network sampling

Twenty-five networks were sampled using the method developed in [31], which consists of a graph Hamiltonian that allows the creation of random networks close to specified nodal degree and clustering coefficient values. Sampling converges to networks with desired specified connectivity (details on the algorithm and implementation can be found in [31]). For sampling, values of *k* and *C* were set so that they matched real social networks described in [28]. For each value of population size (*n =* 30, 50, 100, 200 and 500), five networks were sampled using a target value of *k* so that *k* ∼ *n^β^*^−1^, where *β* was set to a constant value of 1.677 for all population sizes *n*, yielding target mean degree *k* of 10, 14.1, 22.6, 36.1 and 67.1 for population sizes *n* = 30, 50, 100, 200 and 500, respectively. Note that, as *n* increases, so does the number of neighbours that an agent has on average. The value of *β* was set to be the minimum so that an agent has (at least) 10 neighbouring agents for the smallest population size (*n* = 30). The target value of *C* was set to a constant of 0.25 across all sampling—i.e. the invariable value in real social networks, regardless of population size [28].

Twenty networks were sampled each run (one for each population size *n*), from which five were selected that had parameters close to the target values. Results are shown in table 1. All simulations were implemented using R [32].

**Table 1.** Graph connectivity properties: mean connectivity values averaged across the five graphs selected for each value of population size, *n* = 30, 50, 100, 200 and 500 (s.d., standard deviations).

population size *n*	mean *β* in *k* = n^β−1^	mean nodal degree *k*	nodal degree, s.d.	mean clustering coefficient, *C*	clustering coefficient, s.d.
30	1.676	9.9	0.08	0.251	0.007
50	1.685	14.8	0.2	0.256	0.002
100	1.684	23.4	0.089	0.242	0.001
200	1.681	36.9	0.9	0.250	0.001
500	1.655	62.4	2.2	0.246	0.009

### (d) Implementation

A single run of our simulation is composed of many iterations. On a given iteration, each agent ‘utters’ one convention to one of its neighbours, who is randomly picked from the set of all its neighbours in the graph. The convention produced by the agent can be either part of its repertoire (conventions that have been previously generated or learned by the agent) or invented anew. Conventions are divided into two types: Easy and Hard to learn. Each time an agent ‘invents’ a new convention, that convention is randomly defined to belong to one of these two categories with probability 0.5.

We use an extension of the Chinese restaurant stochastic sampling process to model an agent's selection of a convention to generate. The probability of choosing a given convention, *c*, is proportional to the number of *c* tokens that it has previously generated or heard from its neighbours. More precisely, the probability of selecting an already used convention is defined as2.1
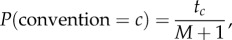
where *t_c_* is the number of tokens of convention *c* that are part of the agent's repertoire and *M* is the number of convention tokens that the agent has stored in memory, thus ∑ *t_c_* = *M*. The probability of inventing a convention anew is defined as2.2
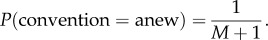


The value of *M* increases over subsequent iterations. However, conventions are eventually lost, either by token forgetting (Poisson forgetting in the horizontal transmission version) or by death of the agent (vertical transmission version). Poisson forgetting is defined at the level of tokens. Each time an agent picks a convention (new or old), then, for each of the *M* convention tokens that it currently stores, there is a probability *p* that this token is ‘forgotten’. This would imply that, on average *M* × *p* tokens are forgotten each time a convention is updated. Given that each time a new convention token is generated, 1 new token is added to M, then M will be in balance when, on average, *M* × *p* = 1. Forgetfulness of tokens captures the idea that cognitive constraints affect the cultural evolution of language [33].

In the vertical transmission version of the model, each time an agent conveys a convention, there is a probability *p* that an agent ‘dies off’—i.e. all the ‘tokens’ in their ‘restaurant’ would disappear. That location in the network would still exist, but is completely cleared, and the ‘dead agent’ is just replaced by a ‘blank slate’ new agent at the same location in the network (like being born into the social network).

Agents can learn conventions from neighbours. The *learned* convention becomes part of the agent's repertoire and can be sampled during its own production. In the current simulations, Easy conventions are defined as those that are learned from only a single exposure, whereas Hard conventions require at least two exposures to be learned.

When an agent uses a convention, to ‘communicate’ with its neighbours, what is the probability that this communication will be successful? We take ‘successful’ communication to imply only that the ‘receiving’ agent also knows that same convention. We are interested in determining the number of Easy and Hard conventions that are successfully used at the population level. Thus, a convention is considered ‘successful’ when it has been learned or generated by one of the agent's neighbours at some point across iterations. Additionally, to get a better sense of successful communication, we measured the proportion of neighbours that share each agent's conventions.

For vertical and horizontal transmission, five separate runs of 1000 iterations were carried out across a range of the parameters *n* (population size) and *p* (probability of forgetting or dying off). At the end of each run, three measures were taken and compared as a function of population size: (i) the (absolute and relative) number of Easy and Hard successful conventions that remained part of the agents' memory (tables in the restaurant), (ii) the (absolute and relative) number of Easy and Hard conventions that remained part of the memory of at least 10% of the agents in the population, and (iii) the mean proportion of neighbours sharing an agent's conventions—that is, for each Easy and Hard convention and for each agent, the proportion of neighbouring agents who had that convention as part of their repertoire was counted. This quantity was averaged over all conventions-agents.

### (e) Results

**Figure 1.**
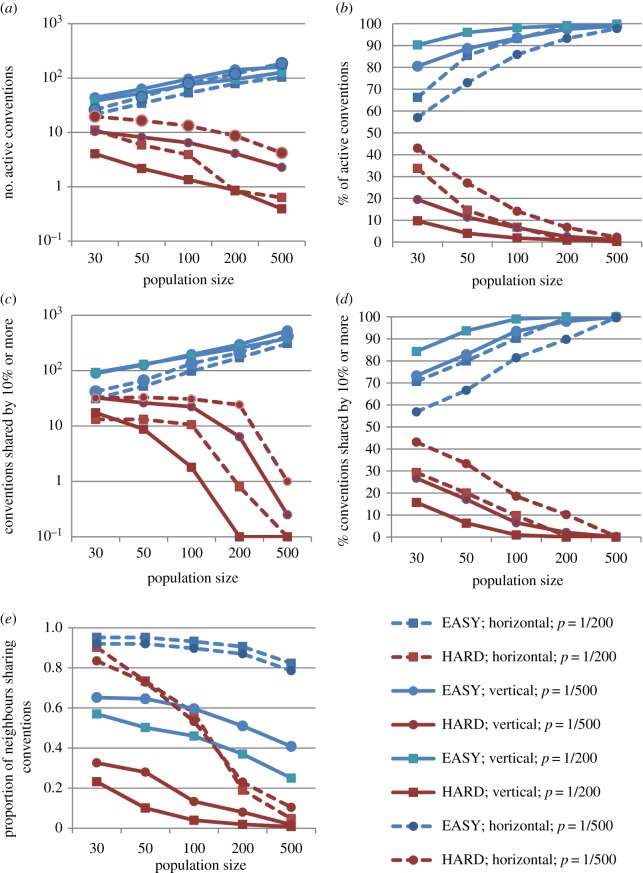
Panels (*a* and *b*) display the results corresponding to the average number of successful conventions per agent—that is, conventions in the agent's repertoire that can be understood by at least one of its neighbours. Panels (*c* and *d*) display the results corresponding to the average number of conventions that are shared by at least 10% of the population. Left panels display absolute numbers (*a* and *c*), and right panels display relative proportions (*b* and *d*) of conventions after 1000 iterations, obtained for increasing values of population size (displayed in the *x*-axis). Panel (*e*) displays the mean proportion of neighbours that share an agent's convention, averaged across all convention-agents. Blue lines correspond to Easy conventions, and red lines correspond to Hard conventions. Dashed lines correspond to results of the horizontal transmission version (circles correspond to the agent's probability of Poisson forgetting *p* = 1/500, while squares correspond to a probability of *p* = 1/200). Solid lines correspond to results of the vertical transmission model (circles correspond to the agent's probability of dying-off *p* = 1/500, while squares correspond to *p* = 1/200).

Absolute and relative values of Hard and Easy conventions after 1000 iterations are shown in figure 1, reflecting a general trend towards an increasing frequency of Easy conventions compared to Hard conventions as the population size increases, in both the vertical and horizontal transmission cases. When the population is small, Hard conventions represent a sizable proportion of the total number of conventions. As population size increases and the overall number of conventions grows, the absolute and relative number of Hard conventions decreases. Both the absolute and relative patterns remain the same across the different conditions, suggesting a robust effect of population size on the proportion of Hard versus Easy to learn conventions. The predictions of the model are, we stress, qualitative: predicting a cross-over between the prevalence of Easy and Hard conventions as population size increases. The population size at which the cross-over occurs depends on parameters, such as the difference between the number of learning trials required for Easy and Hard items (see electronic supplementary material, appendix).

## 3. Discussion

The results suggest that the differential effects of population size on structural complexity and vocabulary size can be accommodated within a parsimonious model of cultural transmission constrained by one cognitive constraint: Ease of Learning. Linguistic innovations that are easy to learn tend to increase in number as a linguistic community grows, because the number of potential innovators increases, and innovations can spread more rapidly. By contrast, small linguistic communities favour linguistic innovations that are hard to learn because they require multiple interactions between individual speakers. It is likely, of course, that many additional forces have shaped the relative development of different aspects of linguistic complexity [2]. One factor that may partly underlie the Easy/Hard distinction considered here concerns the degree to which properties of language can be learned independently. Perhaps an additional reason that learning a lexical item is relatively easy is that word meanings can, to a considerable degree, be learned independently of one another. By contrast, structural aspects of language may interlock in more complex ways, making the propagation of such linguistic innovations more difficult.

More broadly, it is interesting to speculate whether other aspects of cultural evolution may be subject to the pressures described here. For example, perhaps an increase in community size might be associated with a reduction in the prevalence of complex dances, music, rituals, myths or religious beliefs, but an increase in the prevalence of simpler variants (we leave aside skills relevant to survival, such as tool use, whose diffusion will depend on objective measures of efficacy, as well as direct person-to-person contact [34–36]). Of course, such effects may, to some extent, be counteracted by the ability of people to self-assemble into small specialist groups whether face-to-face or virtual, and formal (educational institutions) or informal (salons, discussions groups, artistic movements), to innovate and propagate cultural forms of high complexity. In the absence of the ability for people to self-organize in this way, our simulations raise the possibility that language and culture might become unrelentingly simpler, at the structural level, as human societies become increasingly interconnected.

## Supplementary Material

Additional Variations of Simulation Parameters

## Data accessibility

All source code, data and results are available from: https://github.com/mhchristiansen/lang-paradox.
