# Supplementary material for: Simpler grammar, larger vocabulary: How population size affects language
Source: Proc Biol Sci. 2018 Jan 24;285(1871):20172586. doi: 10.1098/rspb.2017.2586 (PMC5805949; doi:10.1098/rspb.2017.2586)
Supplement: Additional Variations of Simulation Parameters [file rspb20172586supp1.pdf]

Online Appendix for *Simpler grammar, larger vocabulary: How population size affects language*

The predictions of the model are qualitative in nature. The population size at which cross-over between Hard and Easy conventions occurs depends on parameters, such as the difference between the number of learning trials required for Easy and Hard conventions. The graphs below show that Hard conventions tend to decrease over generations while Easy conventions tend to rise for varying values of Hard and Easy learning trials (i.e., number of exposures needed for a convention to be acquired by a new speaker). As shown in the graphs, for varying values of Easy/Hard learning trial ratios, the relative prevalence of Easy conventions over Hard ones depends on population size. This is true for vertical and horizontal transmission and for varying values of dying-off or Poisson forgetting probability.

Percentage of active conventions

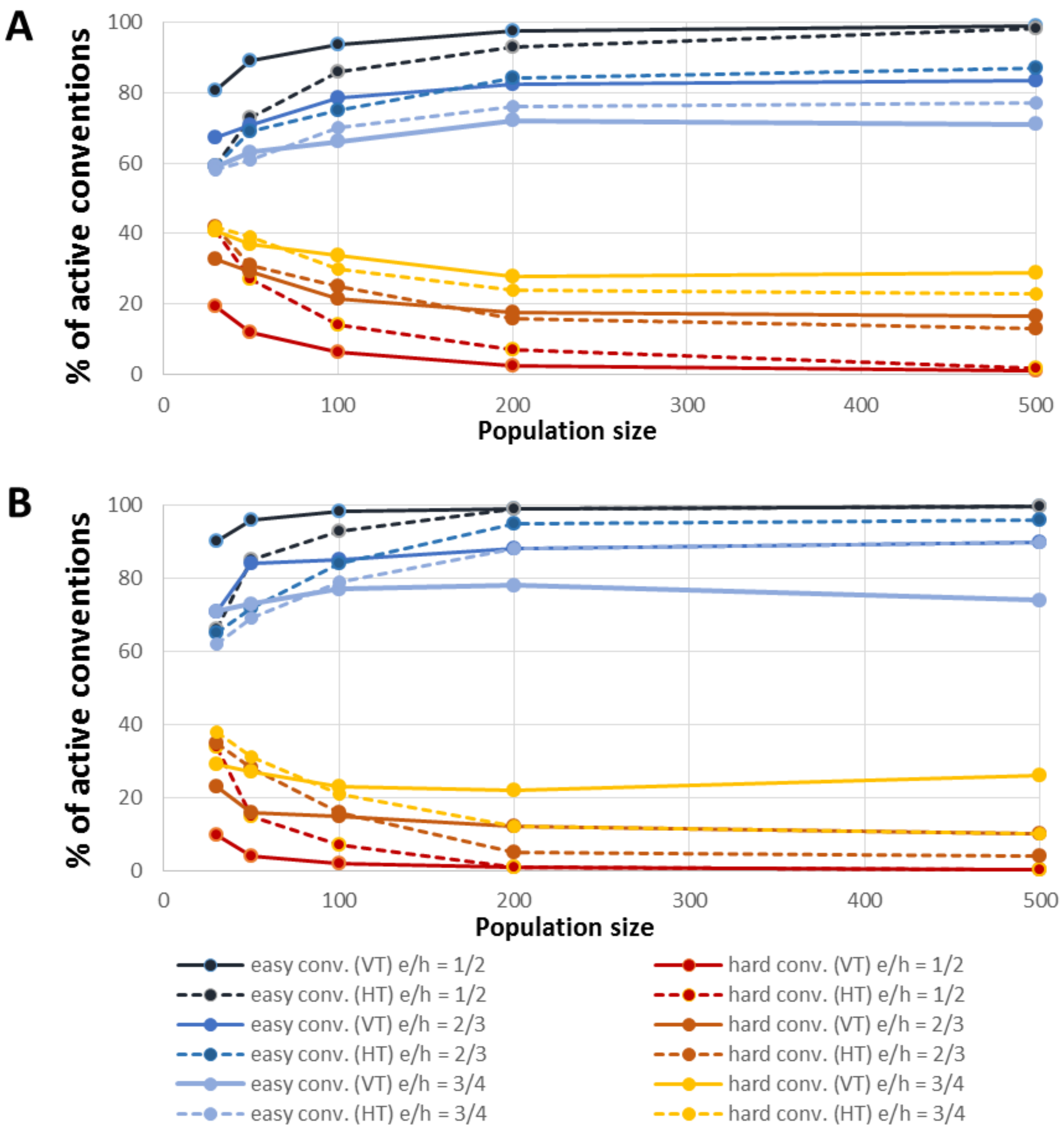

Graph 1. The panels A and B display the average (over 5 simulations) percentage of active conventions for the case of Easy (blue to light blue colors) and Hard (red to yellow colors) conventions after 1000 generations. Results are shown for varying values of Hard and Easy learning trials (i.e., number of exposures needed for a convention to be acquired by a new speaker). The ratios “e/h” displayed in the legends correspond to the ratio of learning trials for Easy (numerator) and Hard (denominator) conventions. Results are shown for the case of vertical transmission (VT; solid lines) and horizontal transmission (HT; dashed lines). The upper panel (A) corresponds to a probability of dying-off or Poisson forgetting $p = 1/500$, while the lower panel (B) corresponds to $p = 1/200$.

Percentage of conventions shared by at least 10% of the population

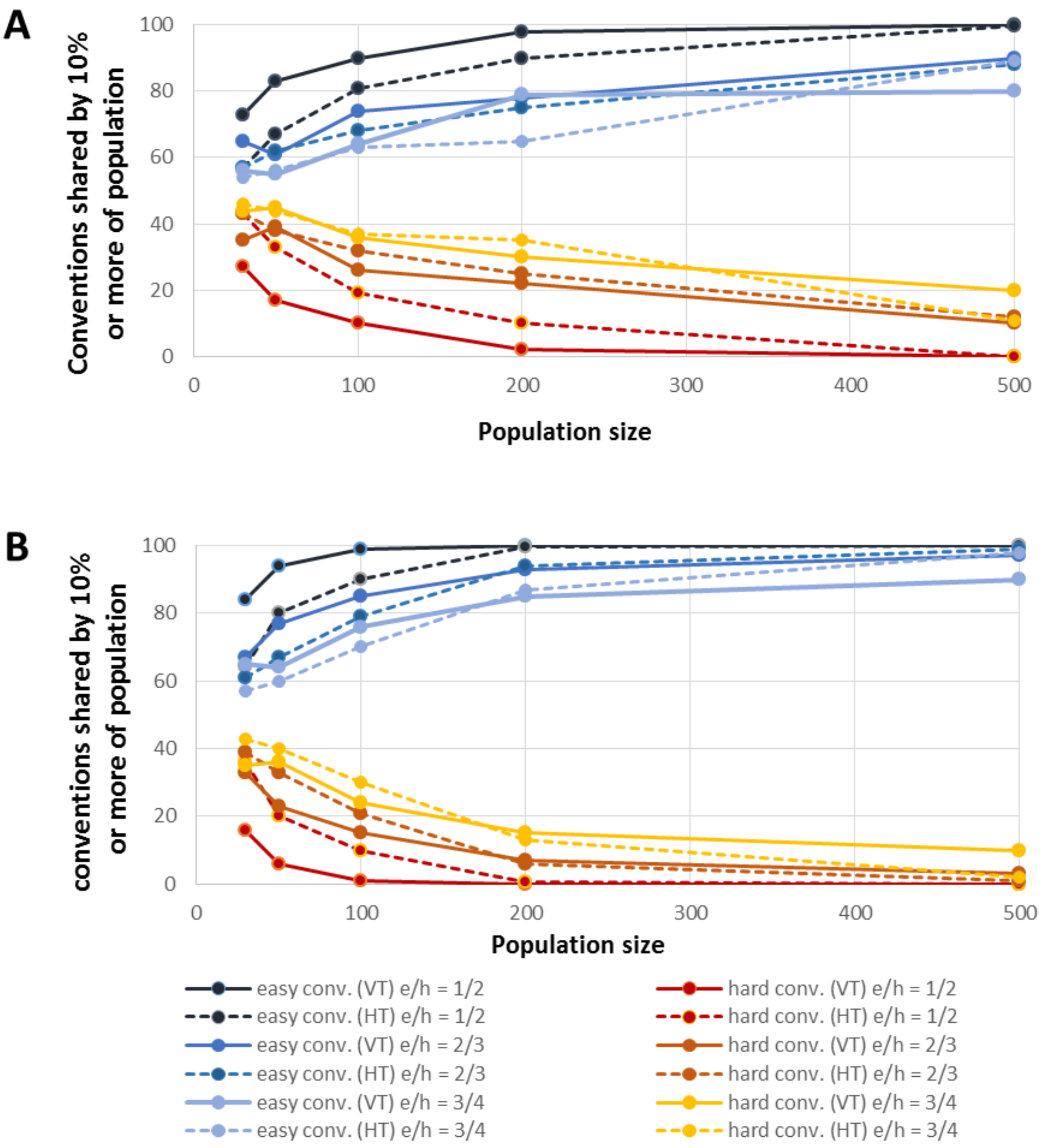

Graph 2. The panels A and B display the average (over 5 simulations) percentage of active convention shared by at least 10% of the population, for the case of Easy (blue to light blue colors) and Hard (red to yellow colors) conventions after 1000 generations. Results are shown for varying values of Hard and Easy learning trials (i.e., number of exposures needed for a convention to be acquired by a new speaker). The ratios "e/h" displayed in the legends correspond to the ratio of learning trials for Easy (numerator) and Hard (denominator) conventions. Results are shown for the case of vertical transmission (VT; solid lines) and horizontal transmission (HT; dashed lines). The upper panel (A) corresponds to a probability of dying-off or Poisson forgetting $p = 1/500$, while the lower panel (B) corresponds to $p = 1/200$.
